# Comparative Analysis of Molecular Detection Algorithms for Enhanced Dengue Surveillance: A Field Case Evaluation

**DOI:** 10.4269/ajtmh.25-0395

**Published:** 2025-12-30

**Authors:** Vidal Felices, Steev Loyola, Crystyan Siles, Roger M. Castillo-Oré, Maria Silva, Julia S. Ampuero

**Affiliations:** ^1^ U.S. Naval Medical Research Unit SOUTH, Lima, Peru;; ^2^ Vysnova Partners, Inc, Alexandria, Virginia

## Abstract

The BioFire® Global Fever Panel (GFP; BioFire Diagnostics, Salt Lake City, UT) is an assay designed for detecting multiple pathogens, including dengue virus (DENV; *Orthoflavivirus denguei*). Although its performance has been primarily assessed using composite reference standards (CRS) or through direct comparisons with other tests, its real-world accuracy remains uncertain. The performance of the GFP in combination with real-time reverse transcriptase polymerase chain reaction (qRT-PCR) testing is evaluated in the present study using dengue surveillance data from 224 individuals with acute fever (≤5 days) in a dengue-endemic area of the Peruvian Amazon. Dengue virus RNA was detected in serum and whole blood using qRT-PCR testing and the GFP, respectively. Two reference standards were developed: one based on CRS and another based on latent class analysis. The best latent class model (LCM) included a viral load surrogate. Using qRT-PCR testing or the LCM as a reference, the GFP exhibited a sensitivity of 94.5% (95% CI: 88.4–98.0%) and a specificity of 75.7% (95% CI: 66.8–83.2%). With CRS, the GFP displayed a sensitivity of 95.6% (95% CI: 90.7–98.4%) and 100% specificity. The simultaneous and sequential two-test algorithms for negative yielded comparable performance and exhibited high sensitivity compared with other two-test sequential algorithms. The sequential testing algorithm, which used qRT-PCR testing followed by the GFP for negative results, offered the best balance in cost, time, and performance (sensitivity: 99.3–100%; specificity: 75.7–100%). Although the GFP is more expensive than qRT-PCR testing, it offers a shorter turnaround time per sample. Overall, the findings suggest that the GFP is a reliable and high-performing tool for DENV testing, but its cost may restrict its use in resource-limited settings.

## INTRODUCTION

Over the past few decades, the global incidence of dengue has increased, causing outbreaks and fatalities in endemic and historically non-endemic areas, including the Americas.^1^^,^^2^ The infection manifests in a wide clinical spectrum, ranging from asymptomatic or mild febrile illness to severe hemorrhagic disease.^3^ Because of its disabling nature, dengue poses a serious threat to the well-being of both military and civilian populations.^1^^,^^3^ Consequently, timely diagnosis during the early stages of infection is essential for effective clinical management, the provision of evidence-based information, and the implementation or improvement of surveillance and control initiatives.

Currently, laboratory assays used for diagnosing dengue include virus isolation, serological methods for detecting dengue-specific immunoglobulins, rapid diagnostic tests (RDTs) such as the nonstructural protein 1 (NS1) antigen-based test, and real-time reverse transcription polymerase chain reaction (qRT-PCR) testing.^3^^–^^5^ Viral isolation requires resources and infrastructure that may not be readily accessible in resource-limited settings and involves labor-intensive procedures with prolonged turnaround times.^3^^,^^6^^,^^7^ Despite the advantages of serological assays, they are potentially susceptible to preexisting antibodies in individuals with secondary infections, presenting challenges for diagnostic use in endemic areas.^4^^,^^6^^,^^8^^–^^10^ The qRT-PCR test provides reliable results more rapidly than viral culture, although it is slower than NS1-based assays. However, its use is restricted to laboratories equipped with the infrastructure necessary to conduct molecular assays, including costly instruments operated by highly trained personnel.^3^^,^^6^ Importantly, none of these assays fully meet the criteria required to be cost-effective, easy to perform, rapid, highly sensitive, and specific regardless of the time of illness or prior infections. Consequently, when negative results are obtained, the reliability of the findings may be compromised. In response to this challenge, the use of combined tests or markers under algorithms has been discussed.^6^^,^^11^^–^^14^

Although empirically managing dengue disease in the absence of laboratory confirmation is common in many dengue-endemic regions, as well as in resource-limited settings or during outbreaks, rapid and reliable positive results in suspected cases would enhance the efficacy of clinical management and potentially reduce costs associated with screening for other febrile illness-causing pathogens.^11^^,^^15^^,^^16^ Rapid diagnostic tests are frequently advantageous in various point-of-care settings, including field surveillance, because of their ease of deployment. However, some RDTs that rely on the detection of *Orthoflavivirus denguei* (dengue virus [DENV]) NS1 or dengue-specific immunoglobulins have exhibited suboptimal performance in evaluations conducted in centralized laboratories.^10^^,^^12^^,^^13^^,^^17^ Although their performance can be affected by intrinsic characteristics of the evaluated population and the study methodology, certain RDTs are prone to cross-reactions with other flaviviruses and residual antibodies from previous DENV infections or other closely related virus infections.^6^^,^^9^^,^^17^ In such circumstances, tests that integrate various advantages of RDTs and detect viral RNA could become a promising diagnostic alternative, particularly in endemic settings that demand fast and reliable results.

The BioFire® Global Fever Panel (GFP; BioFire Diagnostics, Salt Lake City, UT) emerged as a novel assay designed to streamline the automated detection and identification of various pathogens from a single sample using the BioFire FilmArray 2.0 instrument. Novel assays are commonly assessed against a “gold standard” assay, ideally representing a single, error-free reference standard.^18^ However, in practice, this designation is often determined by convention, the “best available” assay, or the “approved” test by regulatory authorities.^18^^–^^20^ Given the absence of a genuine, applicable “gold standard” in real-world scenarios, various approaches have been proposed to mitigate misclassification bias and assess accuracy.^18^^,^^19^ However, to date, the GFP has been characterized by its use of only two approaches, both of which are conducted in a research context: one based on the construction of a composite reference standard (CRS; combined results of tests as a proxy reference) and another based on a side-by-side comparison against a polymerase chain reaction (PCR) test within the context of surveillance for DENV and other fever-causing pathogens.^21^^,^^22^ Therefore, the evaluation of its performance using additional approaches and its potential integration into a testing algorithm alongside another test remains unexplored. The aim for the present study was to characterize the performance of the GFP and compare various two-test algorithms using dengue surveillance results obtained with the GFP and qRT-PCR testing. Both previously established approaches and analyses based on latent class models (LCMs) were used.

## MATERIALS AND METHODS

### Study design.

A retrospective cross-sectional diagnostic accuracy study was conducted.

### Study population.

The study participants were patients aged 5 years and older who presented with an undifferentiated acute febrile illness within 5 days after symptom onset. The presence of fever was ascertained through body temperature measurements (≥38°C, oral, tympanic, or rectal; ≥37.5°C, axillary) or identified in the medical history within 48 hours before participants arrived at military or civilian health facilities. Data from 224 participants were analyzed here.

Participants were recruited from a dengue-endemic area in the Amazon region of Peru between September 1, 2019 and February 29, 2020. Demographic and clinical information were collected from each participant upon voluntary enrollment. Written, signed consent was obtained from participants aged 18 years and older, as well as parents or legal guardians of minors aged 5–17 years. Written assent was obtained from participants between 8 and 17 years of age once parental consent had been granted.

### Specimen collection and molecular testing.

After obtaining consent, venous blood samples were collected in BD Vacutainer potassium ethylenediaminetetraacetic acid and serum tubes (Becton, Dickinson and Company, Franklin Lakes, NJ). All biological specimens were transported to the laboratory within 4 hours under cold chain conditions. Fresh whole blood samples were processed directly using BioFire GFP Research Use Only (RUO) v1.1 on the BioFire FilmArray 2.0 instrument. In contrast, serum specimens were either processed on the day of collection or frozen at −80°C and then processed within a week for RNA extraction and subsequent qRT-PCR testing for DENV RNA. Serum samples used for testing were not subjected to more than one freeze–thaw cycle. Laboratory personnel were blinded to all molecular test results.

Viral RNA was extracted from 140 *µ*L of serum specimens using the QIAamp Viral RNA Mini Kit (Qiagen, Hilden, Germany; Cat. No. 52904), and the molecular detection of DENV RNA was performed using a qRT-PCR test previously described by Johnson et al.,^23^ with some minor modifications. Briefly, 5 *µ*L of RNA was mixed with 5 *µ*L of a master mix containing TaqMan Fast Virus 1-Step Master Mix (Applied Biosystems, Waltham, MA; Cat. No. 4444432) and specific primers and probes. Dengue virus serotype 1 (DENV-1), DENV-3, and DENV-4 were tested in a multiplex reaction, whereas DENV-2 was tested in a singleplex reaction. The DENV-1 probe was labeled at the 5′ end with 6-carboxyfluorescein and at the 3′ end with a black hole quencher 1 (BHQ-1); the DENV-2 probe was labeled with hexachloro-fluorescein and BHQ-1; the DENV-3 probe was labeled with CAL Fluor Red 610 (CAL 610) and BHQ-2; and the DENV-4 probe was labeled with Quasar 670 and BHQ-3. The qRT-PCR cycling program included a reverse transcription pre-amplification step at 50°C for 5 minutes, followed by a denaturation step at 95°C for 20 seconds, and then a 40-cycle amplification step at 95°C for 3 seconds and 60°C for 30 seconds. Amplification and detection were performed on the Applied Biosystems 7500 Real-Time PCR System, and a positive result for DENV-1, -2, -3, or -4 was determined if the cycle threshold (Ct) value was less than 34. Positive and negative controls were included in every run.

The BioFire GFP RUO v1.1 (Cat. No. DFA2-ASY-0) is a fully automated sample-to-answer assay that detects a panel of six bacterial, four protozoan, and nine viral targets (DENV is included, although the serotype is not reported) directly from a 200 *µ*L whole blood sample in ∼1 hour, requiring 5 to 7 minutes of hands-on time based on experience. This assay encompasses nucleic acid purification from the unknown sample, reverse transcription, high-order nested multiplex PCR, and amplicon melt curve analysis.^24^ Using end melting curve data, the BioFire FilmArray software automatically generates a result for each target.^25^ All procedures were performed according to the manufacturer’s instructions. A comprehensive and detailed description of the manufacturer’s instructions, including performance characteristics, has been previously described.^25^ For the present study, only DENV detection data are reported and analyzed.

### Index tests.

The GFP was considered the index test. Initially, the performance of the GFP was assessed using qRT-PCR as a reference. Subsequently, both the GFP and qRT-PCR were used to construct two reference standards.

### Reference standards.

In the present study, two approaches were implemented to address potential misclassification of disease status resulting from imperfect tests: the CRS and LCMs.^19^^,^^26^^,^^27^ The CRS was constructed to classify individuals using the “OR” rule, as described elsewhere,^26^^,^^28^ assuming that none of the molecular assays yielded false positive results. Then, the statistical modeling method used in the LCMs assumed conditional independence.

In the CRS, individuals were classified as dengue cases if any molecular assay yielded a positive result, whereas those with negative results across all assays were classified as non-cases. Regarding the modeling approach, latent class analysis (LCA) was used to compute the likelihood that each individual was classified as a case or non-case of dengue on the basis of the results observed in each molecular assay.^27^^,^^29^ Specifically, a total of eight LCMs were built: four using a basic two-class model (LCM-1 to LCM-4) to classify individuals into two exclusive and exhaustive groups (case or non-case), and four using a three-class model (LCM-5 to LCM-8) for exploratory purposes and to assess model fit. Considering the influence of viral load and days of illness on the test results,^30^^–^^32^ Ct values (as a surrogate for viral load) were incorporated into LCM-2 and LCM-6 as a covariate, whereas days of illness were included in LCM-3 and LCM-7. Both covariates were included in LCM-4 and LCM-8 to capture their combined effect.

### Sample size calculation.

In this exploratory study, data that were originally not collected were analyzed to characterize the performance of the GFP and molecular detection algorithms. However, using the information outlined in the present study and information described elsewhere,^21^^,^^22^ the sample size was assessed using various calculations,^33^ considering a 5% type 1 error, a 10% marginal error, and a minimum power of 80%. Across all scenarios,^21^^,^^22^ 1) utilizing previously defined parameters in a formula for comparing the GFP with a CRS in a population with known disease prevalence; 2) using the performance of the GFP estimated using the CRS and LCA outlined herein to compare it against previously established characterizations; and 3) using a formula for comparing two tests in a paired design, with information described herein and elsewhere, the computed sample size was smaller or comparable to that used in the current study.

## STATISTICAL ANALYSES

Patient characteristics, hospitalization status, day of illness (calculated as the difference between the symptom onset date and the sample collection date), and DENV test results via qRT-PCR testing and the GFP were described using frequencies. Cycle threshold values were described using medians (p50), and differences were evaluated using the Mann–Whitney *U* test. The agreement between the two molecular assays was assessed using the Kappa statistic. Initially, sensitivity, specificity, and their corresponding 95% exact binomial CIs for the GFP were estimated using qRT-PCR as the reference. Later, the analysis was stratified by patient characteristics, hospitalization status, and day of illness to explore potential variations.

Latent class analysis with and without regressors was conducted using probit models.^19^ The selection of the LCA-derived reference was guided by the log-likelihood, Akaike’s information criterion, and adjusted Bayesian information criterion.^34^^,^^35^ The classes derived from the selected models and CRS groups, labeled according to disease status, were summarized by frequency and then compared with the results of molecular testing. Next, the performance of qRT-PCR or the GFP, as well as their simultaneous or sequential use (confirmatory testing for negatives and positives), was computed using CRS and LCM approaches. Additionally, the performance of qRT-PCR testing and the GFP in relation to days of illness and reference values was assessed. The McNemar’s χ^2^ test was used to assess disparities in classification, and the Kappa statistic was used to assess agreement.

Analyses were conducted in Stata v17 (StataCorp LLC, College Station, TX) and R/RStudio v2022.07.2 for Windows (R Foundation, Vienna, Austria). Significance was defined as *P* <0.05.

## RESULTS

### Population characteristics and dengue testing.

The study population was similarly distributed between males and females; 83.0% were aged 36 years or younger, and most participants had not been hospitalized at enrollment (Table 1). According to the study protocol, all specimens were collected during the acute phase of the disease (1 to 5 days post-onset of symptoms), predominantly within the initial 3-day period (Table 1).

**Table 1:** Patient characteristics and results of DENV testing (*N* = 224)

Variable	*n* (%)
Sex	
Female	102 (45.5)
Male	122 (54.5)
Age (years)	
p50 (min–max)	22 (5–74)
≤18	74 (33.1)
>18–36	112 (50.0)
>36–54	30 (13.4)
>54	8 (3.5)
Hospitalized	
No	207 (92.4)
Yes	17 (7.6)
Day of illness	
1	54 (24.1)
2	60 (26.8)
3	59 (26.4)
4	31 (13.8)
5	20 (8.9)
Serotype-specific qRT-PCR	
Negative	115 (51.3)
Positive*	109 (48.7)
Global fever panel	
Negative	93 (41.5)
Positive	131 (58.5)
Composite reference standard	
Non-case	87 (38.8)
Case	137 (61.2)

DENV = dengue virus; qRT-PCR = real-time reverse transcription polymerase chain reaction.

* 96 DENV-1 and 13 DENV-2.

**Figure 1.**
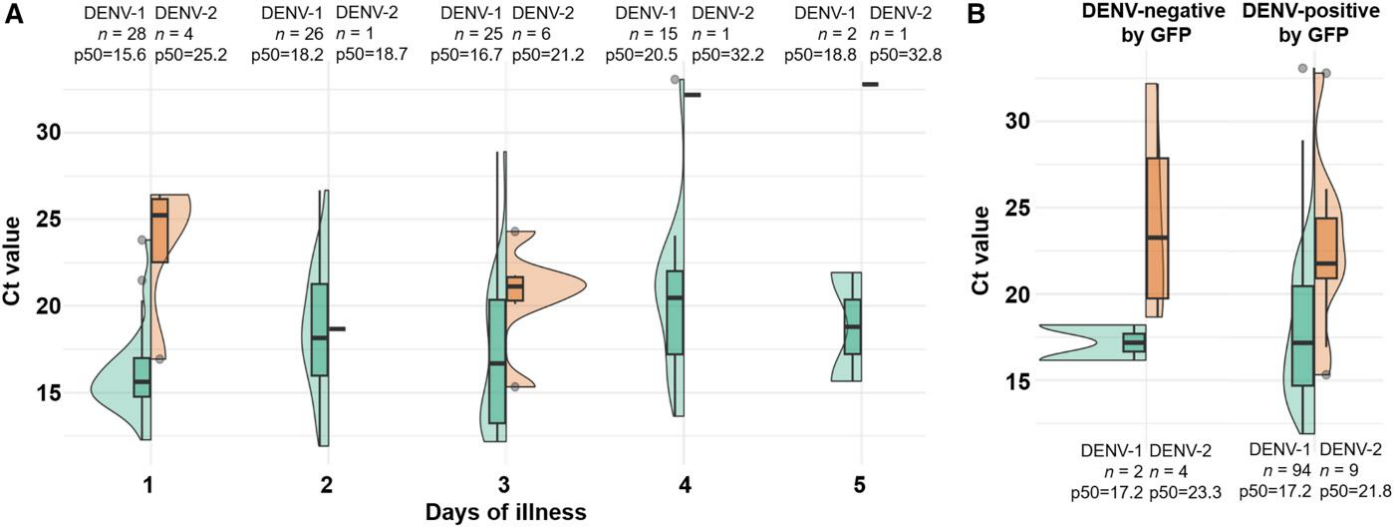
Cycle threshold (Ct) values stratified by illness days and dengue virus serotype, according to Global Fever Panel (GFP) results. The distribution of Ct values is presented using violin and box-and-whisker plots. Cycle threshold values (as a surrogate for viral load) were summarized using sample size and median (p50) and stratified by days of illness and serotype (**A**), as well as by GFP results (**B**).

A total of 109 (48.7%) serum samples tested positive for DENV via qRT-PCR, whereas 131 (58.5%) whole-blood samples were identified as DENV-positive via the GFP (Table 1). The positivity rate was significantly higher with the GFP compared with qRT-PCR testing (*P* <0.001). The qRT-PCR test enabled differentiation of serotypes in serum samples; 96 were DENV-1 (range of Ct values: 11.9–33.1), and 13 were DENV-2 (range of Ct values: 15.3–32.8), among the 109 cases. The Ct values by serotype and days of illness are shown in Figure 1A. Briefly, the median Ct value for DENV-1-positive individuals (p50 = 17.2; *n* = 96) was significantly lower than that for DENV-2-positive individuals (p50 = 21.8; *n* = 13; *P* <0.001). Compared with the first 3 days of illness (p50 = 16.6; *n* = 79), the median Ct value was significantly higher on days 4 and 5 (p50 = 20.8; *n* = 17; *P* = 0.006), particularly in DENV-1-positive individuals.

### Global Fever Panel performance using qRT-PCR as a reference.

The Kappa agreement between the qRT-PCR and GFP assays was 0.698 (95% CI: 0.606–0.790). Among participants, 103 were classified as DENV-positive, and 87 were classified as DENV-negative by both molecular assays (Table 2). On the other hand, 28 “false-positive” results and 6 “false-negative” results were identified by the GFP. In the “false-positive” group, no amplification curves were detected in the qRT-PCR test. In the “false-negative” group, amplification curves were observed. Specifically, two DENV-1 and four DENV-2 cases had median Ct values of 17.2 and 23.3, respectively (Figure 1B). Notably, all results were validated using internal controls as appropriate for the applied assay.

**Table 2:** Performance of the Global Fever Panel for testing using real-time reverse transcription polymerase chain reaction testing as a reference

Performance by	No. of Observations				%Sen. (95% CI)	%Spe. (95% CI)
	TP	FN	TN	FP		
DENV testing*						
Overall	103	6	87	28	94.5 (88.4–98.0)	75.7 (66.8–83.2)
DENV-1	94	2	–	–	97.9 (92.7–99.7)	N/A
DENV-2	9	4	–	–	69.2 (38.6–90.9)	N/A
Sex						
Female	49	1	41	11	98.0 (89.4–99.9)	78.8 (65.3–88.9)
Male	54	5	46	17	91.5 (81.3–97.2)	73.0 (60.3–83.4)
Age (years)						
≤18	33	3	26	12	91.7 (77.5–98.2)	68.4 (51.3–82.5)
>18–36	54	1	45	12	98.2 (90.3–100.0)	78.9 (66.1–88.6)
>36–54	14	2	10	4	87.5 (61.7–98.4)	71.4 (41.9–91.6)
>54	2	0	6	0	100.0 (15.8–100.0)	100.0 (54.1–100.0)
Hospitalized						
No	99	4	80	24	96.1 (90.4–98.9)	76.9 (67.6–84.6)
Yes	4	2	7	4	66.7 (22.3–95.7)	63.6 (30.8–89.1)
Day of illness						
1	30	2	21	1	93.8 (79.2–99.2)	95.5 (77.2–99.9)
2	26	1	27	6	96.3 (81.0–99.9)	81.8 (64.5–93.0)
3	29	2	23	5	93.5 (78.6–99.2)	82.1 (63.1–93.9)
4	15	1	9	6	93.8 (69.8–99.8)	60.0 (32.3–83.7)
5	3	0	7	10	100.0 (29.2–37.9)	41.2 (18.4–67.1)
Illness period (days)						
1–3	85	5	71	12	94.4 (87.5–98.2)	85.5 (76.1–92.3)
4–5	18	1	16	16	94.7 (74.0–99.9)	50.0 (31.9–68.1)

DENV = dengue virus; FN = false negative; FP = false positive; %Sen. = sensitivity; %Spe. = specificity; TN = true negative; TP = true positive.

* The overall evaluation includes all samples tested, and serotype evaluations were restricted to positive samples.

The overall sensitivity and specificity for the GFP were 94.5% (95% CI: 88.4–98.0%) and 75.7% (95% CI: 66.8–83.2%), respectively (Table 2). Although the GFP did not distinguish among DENV serotypes, its performance was significantly better in DENV-1-positive cases (*P* <0.001; Table 2). Sex, age, hospitalization status, and day of illness did not have a significant effect on the GFP performance (Table 2). The specificity of the GFP decreased as the disease progressed, and this observation correlated with an increase in “false positives” (Table 2).

### Global Fever Panel performance using constructed references.

According to the CRS, 137 individuals were classified as DENV cases, and 87 were classified as non-cases (Table 1). Goodness-of-fit indicators and model differences are outlined in Table 3. The models identified as having better indicators included LCM-8, LCM-2, and LCM-4 (Table 3). Latent class model 8, featuring Ct values and days of illness as regressors, facilitated the identification of three distinct classes; notably, class 2 comprised the group of 28 false positives (Tables 3 and 4). Conversely, LCM-2 and LCM-4 differed by one regressor and exhibited comparable indicators (Table 3). Considering the indicators of LCM-3 and the limited influence of days of illness, LCM-2 was preferred over LCM-4 and LCM-8 as the more parsimonious model. On the basis of LCM-2, 109 individuals were classified as DENV cases, and 115 were classified as non-cases (Table 4). Henceforth, the CRS and LCM-2 were considered as the reference standards in subsequent analyses.

**Table 3:** Goodness-of-fit indicators in latent class analysis

LCMs	LL(model)	AIC	aBIC	Class 1, *n* (%)	Class 2, *n* (%)	Class 3, *n* (%)
(LCM-1) two-class	−242.25	494.49	511.55	121 (54.0)	103 (46.0)	−
(LCM-2) two-class + load	−87.06	182.12	195.77	115 (51.3)	109 (48.7)	−
(LCM-3) two-class + days of illness	−239.46	490.92	511.39	115 (51.3)	109 (48.7)	−
(LCM-4) two-class + load + days of illness	−87.06	182.12	195.77	115 (51.3)	109 (48.7)	−
(LCM-5) three-class	−242.25	496.49	516.96	87 (38.8)	34 (15.2)	103 (46.0)
(LCM-6) three-class + load	−112.28	239.16	263.04	87 (38.8)	34 (15.2)	103 (46.0)
(LCM-7) three-class + day of illness	−229.13	478.26	512.37	85 (38.0)	23 (10.3)	116 (51.7)
(LCM-8) three-class + load + days of illness	−78.43	172.87	200.16	87 (38.8)	28 (12.5)	109 (48.7)

aBIC = Bayesian information criterion; AIC = Akaike’s information criterion; LCM = latent class model; LL(model) = log-likelihood value of the regression model.

**Table 4:** Dengue testing results and classification by reference standards

DENV Testing			CRS		LCM-2		LCM-8		
qRT-PCR	GFP	*n*	Non-Case	Case	Non-Case (class 1)	Case (class 2)	Non-Case (class 1)	Und. (class 2)	Case (class 3)
Neg.	Neg.	87	87	0	87	0	87	0	0
Neg.	Pos.	28	0	28	28	0	0	28	0
Pos.	Neg.	6	0	6	0	6	0	0	6
Pos.	Pos.	103	0	103	0	103	0	0	103

CRS = composite reference standard; DENV = dengue virus; GFP = Global Fever Panel; LCM = latent class model; Neg. = negative; Pos. = positive; qRT-PCR = real-time reverse transcription polymerase chain reaction; Und. = undetermined.

The individual sensitivity of the GFP exceeded that of the qRT-PCR test by 16.0% when utilizing the CRS, whereas the qRT-PCR test exhibited a 5.5% higher sensitivity than the GFP when LCM-2 was used as the reference (Table 5). Both molecular assays exhibited an individual specificity of 100% (95% CI: 95.8–100.0%) under CRS conditions, whereas the specificity of GFP was 24.3% lower than that of qRT-PCR when LCM-2 was used as the reference (Table 5).

**Table 5:** Performance of individual, simultaneous, and sequential testing algorithms

Algorithm	CRS		LCM-2		Cost in USD (fold difference)	Run Time (hour)*	Turnaround Time (hour)^†^
	%Sen. (95% CI)	%Spe. (95% CI)	Sen.% (95% CI)	Sen.% (95% CI)			
Individual							
qRT-PCR	79.6 (71.8–86.0)	100.0 (95.8–100.0)	100.0 (96.7–100.0)	100.0 (96.8–100.0)	7,840 (0.16)	15.4	42.5
GFP	95.6 (90.7–98.4)	100.0 (95.8–100.0)	94.5 (88.4–98.0)	75.7 (66.8–83.2)	41,440 (0.84)	224.0	262.1
Simultaneous testing							
qRT-PCR & GFP	99.3 (96.0–100.0)	100.0 (95.8–100.0)	100.0 (96.7–100.0)	75.7 (66.8–83.2)	49,280 (Ref.)	239.4	304.6
Sequential testing for negatives							
qRT-PCR & GFP	99.3 (96.0–100.0)	100.0 (95.8–100.0)	100.0 (96.7–100.0)	75.7 (66.8–83.2)	29,115 (0.59)	130.4	177.0
GFP & qRT-PCR	99.3 (96.0–100.0)	100.0 (95.8–100.0)	100.0 (96.7–100.0)	75.7 (66.8–83.2)	44,695 (0.91)	230.4	279.7
Sequential testing for positives							
qRT-PCR & GFP	73.7 (65.5–80.9)	100.0 (95.8–100.0)	73.7 (65.5–80.9)	100.0 (95.8–100.0)	28,005 (0.57)	124.4	170.0
GFP & qRT-PCR	75.9 (67.9–82.8)	100.0 (95.8–100.0)	94.5 (88.4–98.0)	100.0 (96.8–100.0)	46,025 (0.93)	233.0	286.9

CRS = composite reference standard; GFP = Global Fever Panel; LCM = latent class model; qRT-PCR = real-time reverse transcription polymerase chain reaction; %Sen. = sensitivity; %Spe. = specificity.

* Estimated time for processing 29 samples with qRT-PCR in a single run. In contrast, the GFP processes one sample per hour/machine.

^†^ For qRT-PCR, the total time for 29 samples includes 2.5 hours for RNA extraction, 0.5 hours for reagent preparation, 2 hours for test execution, and 0.5 hours for result analysis. For the GFP, the time includes 0.12 hours (7 minutes) for sample preparation, 1 hour for test execution, and 0.05 hours (3 minutes) for report reading and validation.

**Figure 2.**
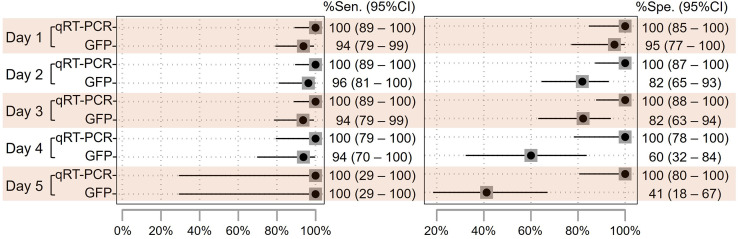
Performance of real-time reverse transcription polymerase chain reaction testing and the Global Fever Panel according to latent class model 2 and illness day.

The selection of LCM over a CRS has been previously discussed.^19^^,^^26^^,^^27^ Herein, LCM-2 was preferred because it allows for the classification of individuals using probabilities in the absence of a gold standard, thereby addressing uncertainty about the “real” disease status. By stratifying the performance of each molecular assay using LCM-2 by days of illness, the qRT-PCR test and the GFP exhibited similar sensitivity, but the GFP exhibited reduced specificity during the fourth and fifth days of illness (Figure 2).

### Two-test algorithms for DENV detection.

In 2020, the estimated cost of running qRT-PCR testing (including RNA extraction [$8.00], PCR reagents [$15.00; two reactions for DENV testing and one for RNase P detection], and supplies [$12.00]) at the NAMRU SOUTH headquarters laboratory in Lima, Peru, was $35.00 per sample, whereas the cost of the GFP assay (one pouch with freeze-dried reagents [$183.00] and supplies [$2.00]) was $185.00 per sample. These estimates exclude equipment and labor costs. The qRT-PCR test can process up to 29 samples simultaneously in ∼2 hours, detecting any of the four DENV serotypes. In contrast, the GFP processes only one sample at a time and identifies DENV without differentiating by serotype. Although qRT-PCR costs can be reduced by using serotype-indiscriminate techniques, the GFP has the advantage of detecting multiple non-DENV pathogens and coinfections. All this information was used as a reference for the analyses performed. The costs, run times, and turnaround times from nucleic acid extraction to result analysis are summarized in Table 5.

Among the evaluated two-test algorithms, the simultaneous two-test algorithm was the most expensive and time-consuming, whereas the sequential testing algorithm for positives based on qRT-PCR followed by the GFP was the least costly and time-consuming (Table 5). The study findings suggest that the sequential testing algorithm for negatives based on qRT-PCR followed by the GFP provided the best balance of performance, cost, and time (Table 5).

## DISCUSSION

The dramatic increase in DENV cases over recent years has exceeded the diagnostic capacity in resource-constrained endemic regions of the Americas, highlighting the urgent need for improved testing tools.^3^^,^^7^^,^^36^^–^^38^ Laboratory diagnosis of suspected dengue relies on various techniques to confirm infection.^3^^–^^5^ However, each recommended assay presents critical challenges related to laboratory capacity or capabilities, cost, availability of instruments, ancillary equipment and consumables, the need for well-trained personnel, sample collection timing, and performance.^6^^,^^7^ Given these challenges and limitations, there is still a need to assess emerging molecular tools that are automated, deployable, and do not require highly trained operators. The GFP has emerged as a promising molecular tool for dengue testing; however, to the authors’ knowledge, its evaluation in real-world and endemic contexts remains scarce and limited to two previous reports that relied on samples from sub-Saharan Africa, Southeast Asia, Central and South America, the United States, and Europe.^21^^,^^22^ In the current study, using information derived from DENV investigation and surveillance in a Peruvian Amazon region with high endemicity and co-circulating serotypes, the performance of the GFP is reported using qRT-PCR, a CRS, and LCM-2 as references. The overall performance of simultaneous and multiple sequential algorithms is also described using the GFP and qRT-PCR testing.

In the present study, 34 discrepancies were noted between qRT-PCR and the GFP. Of these, 28 individuals tested negative via qRT-PCR but positive via the GFP. A similar finding, although at a lower degree, has been reported previously by Camprubí-Ferrer et al.^22^ This observation could be explained not only by differences in detection thresholds between assays but also by pre-analytical factors, including the use of different sample matrices (whole blood for the GFP versus serum for the extraction of RNA used in qRT-PCR), which could influence viral RNA recovery and downstream detection. Conversely, six individuals tested positive via qRT-PCR but negative via the GFP, a result that has also been documented.^21^^,^^22^ Manabe et al.^21^ suggested that in such instances, misclassification can potentially be corrected by either repeating the GFP or conducting additional molecular assays. In the present study, the GFP was not repeated, and given the reliability of the qRT-PCR results (as indicated by amplification curves, comparable Ct values with other positive samples, and no evidence of contamination), no additional tests were performed. Overall, the absence of a third reliable testing method prevented the full resolution of discrepancies. Future investigations using matrix-matched panels of well-characterized positive specimens (via molecular and serological assays) and negative specimens (including those from nonendemic regions) will be critical to further validate and refine the study findings.

The sensitivity and specificity of the GFP, when compared with a molecular reference, have been reported as 80.8–94.0% and 97.9–100.0%, respectively.^21^^,^^22^ Comparable performance was observed when using an NS1-based RDT as a reference.^22^ In the present study, although methodological differences exist and qRT-PCR was used as a reference, the GFP sensitivity (94.5%; 95% CI: 88.4–98.0%) was consistent with previous reports. However, its specificity (75.7%; 95% CI: 66.8–83.2%) was lower than reported elsewhere.^21^^,^^22^ When the performance of the test was analyzed by days since illness onset, the specificity for the first 3 days was higher and similar to that reported previously.^21^^,^^22^ Therefore, the lower global specificity reported here may be influenced by the inclusion criteria.^32^ However, it is essential to highlight that direct comparisons between two imperfect tests are not recommended.^19^^,^^26^^,^^27^ Consequently, performance metrics derived from side-by-side comparisons should be interpreted with caution.

Camprubí-Ferrer et al.^22^ previously assessed GFP using a CRS comprising both direct (RDT and PCR) and indirect (serology) assays, yielding sensitivity and specificity values of 68.5% (95% CI: 57.1–78.0%) and 100.0% (95% CI: 98.8–100.0%), respectively. In the present study, using a CRS based only on molecular testing, a higher sensitivity (95.6%; 95% CI: 90.7–98.4%) and comparable specificity (100.0%; 95% CI: 95.8–100.0%) were observed. The variation in sensitivity may be attributed to differences in the composition of the CRS.^20^ Specifically, although integrating both direct and indirect tests typically enhances accuracy, serological tests may contribute less, particularly in cases of acute disease.^3^^,^^4^ Using LCM-2 as a reference, the sensitivity of the GFP was comparable with that estimated by the CRS used in the current study and higher than that described elsewhere.^22^ However, the specificity (75.7%; 95% CI: 66.8–83.2) was notably lower than that calculated using the CRS. This reduced specificity is attributable to the classification of 28 individuals as non-cases on the basis of their qRT-PCR-negative and GFP-positive results. Incorporating additional tests could improve reference standards and enhance discrimination and classification in future studies. However, on the basis of all observations and regardless of whether a CRS or LCM-2 is used, the study results consistently suggest that the GFP is an assay with acceptable sensitivity and specificity.

The results indicate that qRT-PCR and the GFP exhibit comparable performance during the first 3 days of illness. However, the specificity of the GFP evidently decreases after 4–5 days, likely because higher Ct values reflect lower viral loads as the infection progresses, making it less suitable for use beyond this period. Incorporating both the GFP and qRT-PCR into testing algorithms could significantly enhance case detection. Whether using CSR or LCM-2 and regardless of the testing order, both simultaneous and sequential testing for negatives yielded similar overall performance, notably maximizing sensitivity, as previously described.^14^^,^^39^ Among these two algorithms, sequential testing for negatives starting with qRT-PCR, followed by the GFP, was the most cost-effective and had the shortest run and turnaround times. Conversely, sequential testing for positives may improve overall specificity when there is a high suspicion of false positives from the initial test. Building on these findings, the authors of future studies should explore whether replacing qRT-PCR with RDTs could further advance the development of deployable and flexible algorithms that operate independently of centralized resources.

The present study has some additional limitations. First, serotype-specific analyses revealed high sensitivity for DENV-1. Although the sensitivity for DENV-2 was acceptable, the limited number of cases for this serotype should be acknowledged. The authors of previous studies did not evaluate serotype-specific sensitivity, likely because the GFP was unable to differentiate between serotypes.^21^^,^^22^ Nonetheless, it is plausible that viral mutations within specific serotypes and other non-studied factors could affect performance, as previously described.^40^ Second, the present study’s non-multicenter design differed from the two previous studies,^21^^,^^22^ yet the findings align with those studies. Third, several factors that could influence performance were not fully characterized or included in the analyses. These factors include the presence of symptoms, illness duration, and preexisting immunity against DENV or other flaviviruses, which affect viral clearance and, consequently, assay performance.^6^^,^^10^^,^^32^ Future research with larger sample sizes and varied viral diversity is needed to better understand how these factors affect the performance of the GFP in dengue surveillance. The current study has four notable strengths: a sufficient sample size to ensure reliable and precise estimates, the use of LCA as a novel methodological advance that was not previously used,^21^^,^^22^ stratified analyses to assess robustness, and the potential application of the GFP in various testing algorithms to optimize performance.

## CONCLUSION

In summary, the study results reveal that the GFP is a reliable and effective tool for DENV detection, whether used as a single test or integrated into a testing algorithm. Researchers conducting future evaluations should address the cost-effectiveness of its implementation in various epidemiological and economic contexts. For surveillance purposes in dengue-endemic areas, the study findings indicate that the overall sensitivity or specificity can be optimized within a testing algorithm, while also reducing costs and turnaround times.
